# Optimizing Drug Response Study Design in Patient-Derived Tumor Xenografts

**DOI:** 10.1177/11769351221136056

**Published:** 2022-11-22

**Authors:** Jessica Weiss, Nhu-An Pham, Melania Pintilie, Ming Li, Geoffrey Liu, Frances A Shepherd, Ming-Sound Tsao, Wei Xu

**Affiliations:** 1 Department of Biostatistics, Princess Margaret Cancer Centre, University Health Network, University of Toronto, Toronto, ON, Canada; 2 Princess Margaret Cancer Centre, University Health Network, Toronto, ON, Canada; 3 Department of Medicine, Division of Medical Oncology, University of Toronto, Toronto, ON, Canada; 4 Department of Medical Biophysics, University of Toronto, Toronto, ON, Canada; 5 Department of Laboratory Medicine and Pathobiology, University of Toronto, Toronto, ON, Canada; 6 Department of Biostatistics, Dalla Lana School of Public Health, Toronto, ON, Canada

**Keywords:** PDX, xenograft, replicates, treatment effect size, lung cancer, therapy response, mixed-effect model, tumor growth rate, 1 × 1 ×1, n = 1 experiment

## Abstract

Patient-derived tumor xenograft (PDX) models were used to evaluate the effectiveness of preclinical anticancer agents. A design using 1 mouse per patient per drug (1 × 1 × 1) was considered practical for large-scale drug efficacy studies. We evaluated modifiable parameters that could increase the statistical power of this design based on our consolidated PDX experiments. Real studies were used as a reference to investigate the relationship between statistical power with treatment effect size, inter-mouse variation, and tumor measurement frequencies. Our results showed that large effect sizes could be detected at a significance level of .2 or .05 under a 1 × 1 × 1 design. We found that the minimum number of mice required to achieve 80% power at an alpha level of .05 under all situations explored was 21 mice per group for a small effect size and 5 mice per group for a medium effect size.

## Introduction

Cancer incidence and mortality continue to increase worldwide; 1 in 5 men and 1 in 6 women will be diagnosed with cancer in their lifetime.^1,2^ Patient-derived tumor xenografts (PDX) have proven to be useful models in studying human cancer. They have been shown to reflect more accurately the characteristics of patient tumors when compared to cell line models.^3,4^ PDX models are generated by implanting patient tumor fragments or cells directly into immune-deficient mice and propagated serially to allow for preclinical drug studies.^5^ Mice replicates carrying the same PDX model can be tested for different drugs or drug combinations, their mechanisms of action and for biomarker discovery. However, to perform this research, each PDX model has to be classified accurately, according to the degree of drug efficacy in affecting tumor growth. The first PDX study was reported in 1969 and in recent years the use of PDX models has grown in popularity.^6,7^

**Figure 1.**
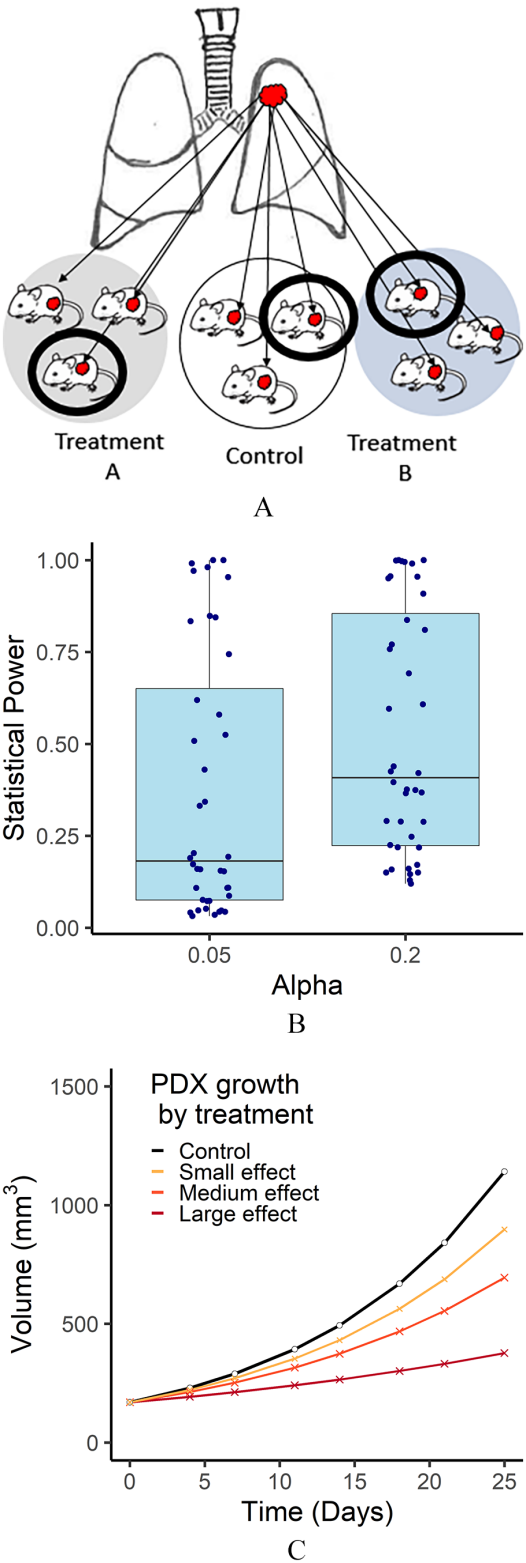
(A) A PDX study design to compare 2 treatments to a control. Larger circles illustrate a study with 3 mice per treatment. Thick black circles illustrate a 1 × 1 × 1 design with 1 mouse per treatment. (B) Estimated statistical power based on real individual experiments. Power is calculated based on a 1 × 1 × 1 PDX design for alpha levels (type I error rates) of .05 and .2. (C) Categories of growth effects. Examples of growth rates for a mouse given a control, and drugs of a small, medium, and large effect size. The control growth rate is based on the median control of the observed data, and effect sizes are divided at the quartiles of the observed data.

While PDXs are good pre-clinical models for studying cancer biology and interventions, there are some limitations. These include the lack of host immune system,^8^ engraftment failure, variation among mouse replicates for individual models, and relatively long PDX growth duration (1-4 months).^5^ The number of mouse replicates per treatment group has typically ranged between a few to a dozen mice.^9-12^ To reduce the number of mice in PDX experiments (and improve costs and logistics), a 1 mouse per model per treatment (1 × 1 × 1) design has been utilized (Figure 1A). The 1 × 1 × 1 approach was first proposed by Migliardi et al^13^ and was extensively applied by Gao et al^14^ in a preclinical cancer drug study using 1000 PDX models.

There is currently no consensus on the statistical method to assess PDX tumor growth and a variety of methods have been utilized.^15^ One popular method is assessing the tumor growth inhibition by comparing the tumor volumes at a specified time or the end of the experiment.^16-19^ Similarly, comparing the mean doubling time of the control versus treated has been used.^20^ Both methods are generally straightforward and easily interpretable; however, they do not utilize all tumor growth information. The area under the curve (AUC)^21,22^ utilizes all of the tumor information, but the results are not represented in meaningful units. The mRESIST (modified Response Evaluation Criteria In Solid Tumors) criteria^14,20,23^ is another common approach used to summarize PDX growth. This approach uses a combination of the average response, and best average response to categorize tumors as a complete responder (CR; complete disappearance of the tumor), partial responder (PR; significant shrinkage of the tumor), stable disease (SD; minimal change in tumor volumes), or progressive disease (PD; significant growth of the tumor). There are some limitations of mRESIST, the method is not designed to compare treatments to a control, the magnitude of the treatment effect is not given beyond the categorical thresholds, and no *P*-value is calculated. PDX response has also been studied by comparing the slopes of the tumor growth curves^22^ and by comparing the growth rates for exponentially growing models.^17^ Comparing the slopes of many mice can be performed using linear mixed-effects models (LMM).^24^ Advantages to modeling PDX growth include; utilizing of all mouse-follow-up information, the growth rate estimates are interpretable, and *P*-values are estimated.

Statistical power is defined as the probability of correctly rejecting a null hypothesis when the alternative hypothesis is correct. Statistical power corresponds directly with type II error. Where a type II error occurs when the null hypothesis fails to be rejected when the null hypothesis is incorrect. When designing a study, the goal is to maximize the power while minimizing the alpha (α). Alpha is the probability of a type I error, where a type I error occurs when the null hypothesis is incorrectly rejected. Typically, the null hypothesis of a PDX study is that the treated tumors are growing at the same rate as the controls. Thus, a type II error would occur if there was a difference in tumor growth and the null hypothesis was not rejected. A type I error would occur if there was no difference in tumor growth but the null hypothesis was rejected. The standard when designing a study is to have an alpha level of .05, and power of 80%.^25^

There have been previous works which assessed the statistical power of mouse PDX studies in the 1 × 1 × 1 setting. Gao et al^14^ reported that mRESIST correctly categorized the 1 × 1 × 1 PDX results 66% of the time (based on the most frequent category) and categorized them as responder versus (vs) non-responder in 95% of the time. In another study, Guo et al^20^ ran simulations of an LMM design on a 21-day cisplatin treatment dataset of 42 PDX models and found power to be below the 0.80 thresholds for all values of the treatment effect explored with the 1 × 1 × 1 design for single patient models.

## Methods

### Methodology to establish and measure PDX models

We utilized non-small cell lung cancer (NSCLC) PDX drug response data collected between 2012 and 2015. Animal care followed the guidelines of UHN Research Institutes’ policies and the guidelines of the Canadian Council on Animal Care, and was consistent with ARRIVE guidelines for study design.^26^ The University Health Network (UHN) Human Research Ethics approved this PDX study (REB# 09-0510), following the human research guidelines of the Canada Tri-Council Policy Statement, in accordance with the Declaration of Helsinki (www.pre.ethics.gc.ca.). In each experiment, PDX models were established from patient tumor fragments that were implanted subcutaneously in the flank of non-obese severely combined immune-deficient (NOD SCID) [NOD.Cg-Prkdc<scid> Il2rg<tm1Wjl>/SzJ:(Stock #JAX:5557)] mice.^27^ Xenograft tumor fragments were expanded into mouse replicates to test with anti-cancer agents.^9-11^ Experiments were included based on having no missing or ambiguous values, a minimum of 4 mice per identical treatment group, and at least 4 longitudinal measurements per mouse. In experiments with more than 2 treatment groups, we randomly selected a treatment to compare with the control. For simplicity, we only included the common follow-up time points for all mice within an experiment.

### Linear mixed models

We focused on using the LMM approach, as we hypothesized that log-linear mixed-effects modeling was the optimal method for studying PDX growth. In the case of PDX growth which was often exponential, a log-linear mixed-effects model was utilized^20^ (Supplemental Equation 1). When there was only 1 mouse per group the LMM model reduced to a linear model (Supplemental Equation 2). Our work went further than previous studies^14,20^ by exploring modifications in the effect size, alpha level, across mouse variation, measurement frequency, and follow-up duration, to discover optimal conditions for using a 1 × 1 × 1 approach.

### Condition settings

**Figure 2.**
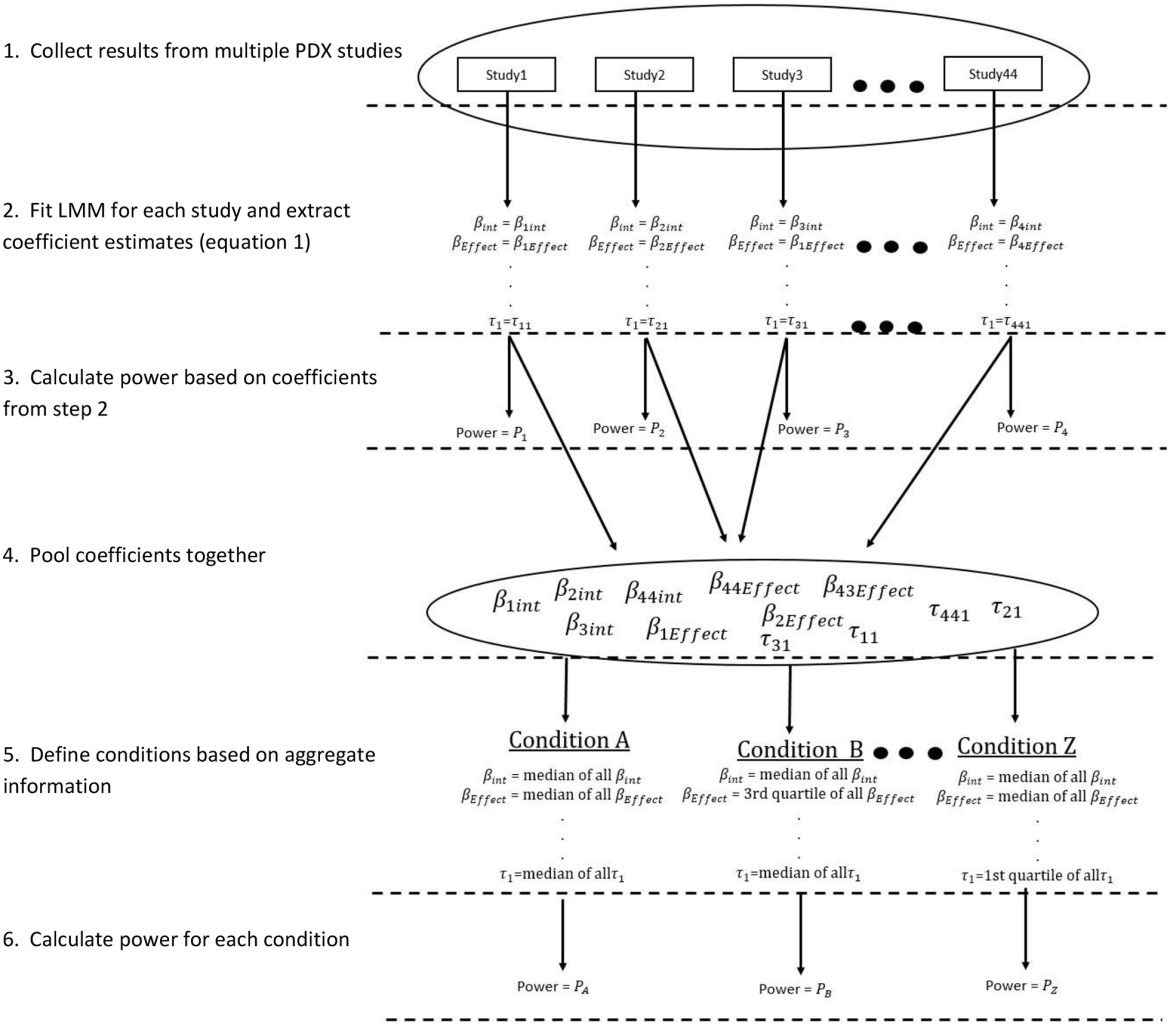
Flow chart demonstrating power calculations.

We estimated statistical power under different condition settings to determine the situations where a 1 × 1 × 1 design was appropriate. The statistical power was calculated using simulations and with a power formula (see Supplemental Methods for more details). For all conditions 2 alpha values were included, the traditional α = .05 and a relaxed α = .2. All conditions were derived from the results of fitting log-linear mixed-effects models for each real experiment (Supplemental Equation 1; Supplemental Table 3). The mixed-effects models were used to acquire coefficient estimates of the baseline tumor sizes, growth rates of the control tumor, effect size, and variation (Figure 2 steps 1-2).

We assessed the statistical power in 3 different ways, firstly by treating each study as a 1 × 1 × 1 study, second by modifying 1 feature over a range while keeping everything else constant, and finally by simultaneously varying different features.

To assess each study as if it had been run as a 1 × 1 × 1 all estimated coefficients and the number of tumor measurements for each experiment were used as conditions (Supplemental Table 3; Step 3 in Figure 2).

In addition to treating each study independently, we also viewed the studies as a whole by using aggregate information of medians and quartiles from all of the studies (Supplemental Figure 2; Supplemental Table 3). We used the medians and quartiles as they were realistic estimates for effect size based on the real data, and allowed us to generate a biologically relevant range of conditions to explore power (Steps 4-6 in Figure 2).

To look at the effects of varying a single feature we created conditions consisting of medians for all coefficients and measured twice weekly for 4 weeks (day 0, 4, 7, 11, 14, 18, 21, and 25), in addition to 1 modification. The modifications included varying the ${\hat{\beta }}_{Effect}$ (the difference in log daily growth of treated vs control), and ${\hat{\tau }}_{1}$ (the inter-mouse variation/variation in growth across mice given the same treatment), the follow-up frequency, and the study duration. These conditions are summarized in Table 1.

**Table 1.** Statistical power conditions with 1 feature varying.

Figure	${\hat{\beta }}_{Effect}$	${\hat{\tau }}_{1}$	Number of weeks measured	Number of times measured per week
3A	20 values evenly from −0.08 to 0.019	0.005	4	2
3B	−0.02	20 values evenly from 0.0001 to 0.02	4	2
3C	−0.02	0.005	1-8	2
3C	−0.02	0.005	1-8	3

**Figure 3.**
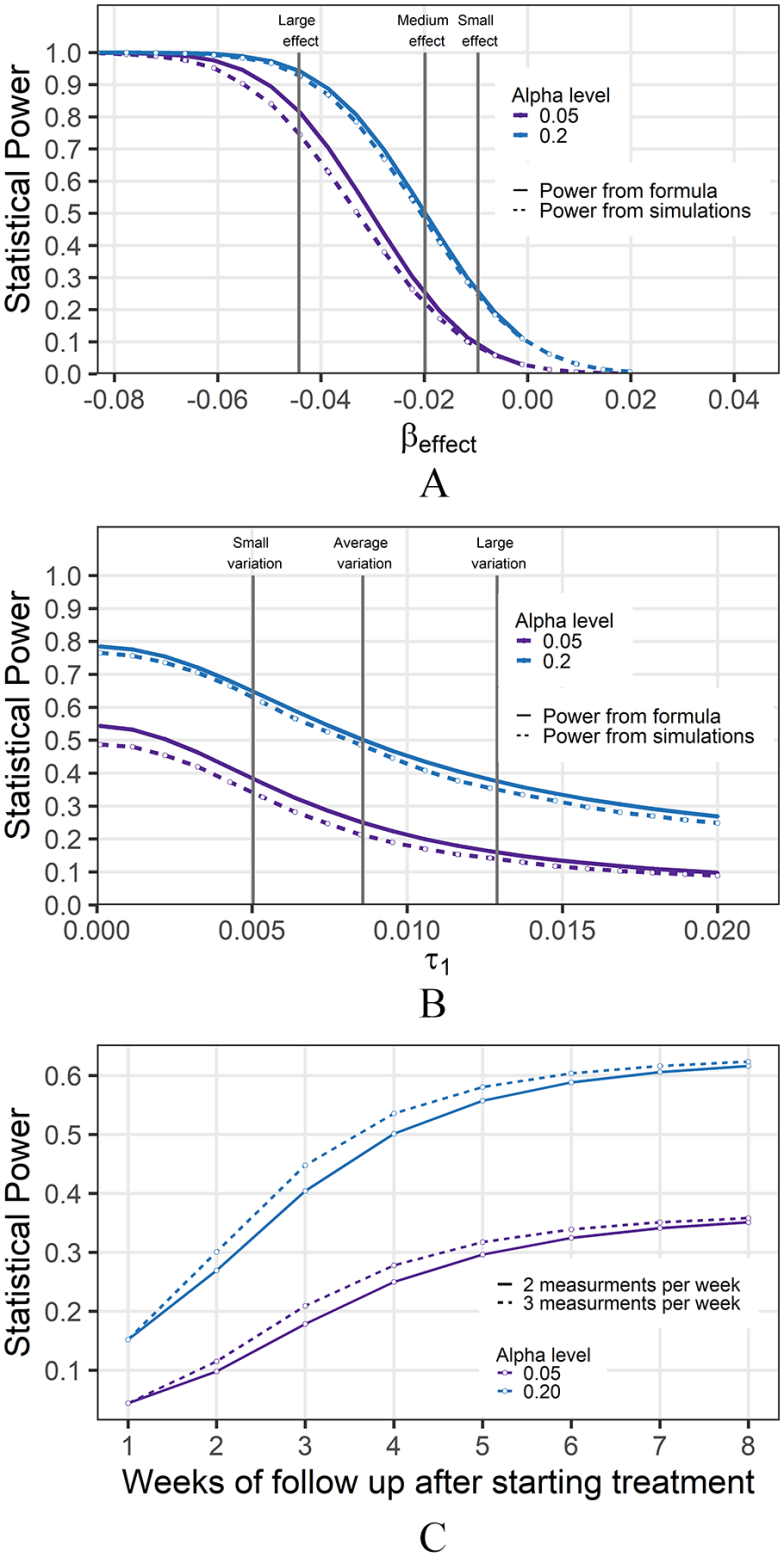
Calculated statistical power under varying conditions. Coefficients were set to the median from all studies and follow-up was set to twice a week for 4 weeks. (A) Treatment effect size relationship with power. Highlighted are large, medium, and small effect sizes based on the quartiles of the treatment effects from combined lung experiments. (B) Mouse variation relationship with power. Highlighted are small and average intra-mouse variations based on the quartiles of the variation from combined lung experiments. (C**)** Measurement frequency and duration relationship with power.

Conditions used for Figure 3A to C with 1 feature being varied.

Finally, we created conditions using combinations of modifications involving ${\hat{\beta }}_{Effect}$ at either the median (medium effect), upper quartile (small effect), or lower quartile (large effect) of the real data. ${\hat{\tau }}_{1}$ at either the median (average variation), or lower quartile (small variation) of the real data, for a duration of 3 or 4 weeks, and measuring twice or 3 times a week (Steps 4-6 in Figure 2). For the combination settings, we rearranged the LMM power formula to also calculate the sample size required in each group to achieve 80% power.

## Results

Cumulative PDX drug efficacy studies used for analyses included 44 NSCLC PDX experiments that tested a total of 14 different anti-cancer agents (Supplemental Tables 1 and 2). Each experiment included an average of 6 replicates per group (531 total mice), from 25 unique PDX models. All data generated or analyzed during this study are included in this published article (and its Supplemental Material).

### Estimated power for each real experiment if it had been run as a 1 × 1 × 1

We investigated each PDX study using results from LMM models which ran independently for each experiment (Supplemental Table 3). Figure 1B illustrates the estimated power of each study if it had been designed as a 1 × 1 × 1 study. Four studies that showed no treatment effect were excluded. In our power calculation, the majority of studies would not have had sufficient power had they been run as a 1 × 1 × 1 study. The estimated statistical power was above 80% only for 9/40 (22.5%) and 12/40 (30%) experiments when alpha was set to .05 and .2, respectively. Additionally, the median estimated power was only 18% when the alpha level was set to .05 and 41% when the alpha level was set to .2.

### Power under aggregate settings with a single variation

Log-linear mixed-effects models were performed on each individual study to obtain parameter estimates (medians based on coefficients in Supplemental Table 3 and Supplemental Figure 1). This included ${\hat{\beta }}_{int}$ = 5.135, ${\hat{\beta }}_{Day}=0.076$ , ${\hat{\beta }}_{Effect}$ = −0.02, $\hat{\sigma }$ = 0.155, γˆ = 0, $\tau_{0}=0.28$ , ${\hat{\tau }}_{1}$ = 0.009, and ${\hat{\tau }}_{01}$ = 0.034. We considered a small effect to be the top quartile of the estimated treatment effect size (v ${\hat{\beta }}_{Effect}$ = −0.010), and a large effect to be the bottom quartile ( ${\hat{\beta }}_{Effect}$ = −0.044). Using the formula $e^{7\times \beta_{Effect}}$ the large, medium, and small ${\hat{\beta }}_{Effect}$ values were equivalent to the treated tumors growing at a weekly rate of 0.73, 0.86, and 0.93 times the weekly growth rate of the control mice (Figure 1C). This means that if a control tumor was doubling every week, a tumor treated with a large effect would increase by 2 × 0.73 or 1.46 times each week. Additionally, when we instigated the inter-mouse variation, we considered a small amount of variation to be the bottom quartile ( ${\hat{\tau }}_{1}$ = 0.005), and a large amount the upper quartile ( ${\hat{\tau }}_{1}$ = 0.013).

### Power and treatment effect size

The calculated and simulated power under different settings of a treatment effect is shown in Figure 3A. We defined large, medium, and small effect sizes based on the quartiles and median of the estimated effect sizes from our real data. When the treatment effect was considered large, power was found to be greater than 80% at both alpha levels of .05 and .2, based on the power calculation formula. The slope of the power curves was very steep between the large treatment and medium effects and indicated a small decrease in the treatment effect can have a large impact on study power. For both alpha levels, the power was determined to be under 80% for medium and small treatment effects.

### *Power and* inter-mouse variation

The calculated and simulated power under different mouse growth variations is shown in Figure 3B. The power was well below the 80% threshold when alpha was set to both .05 and .2 for small, average, and large variation. Additionally, the curve was not very steep between the small and average variation, indicating that the variation in this context did not play a large role in the determination of power.

### Power and follow-up schedule

There was an increase in power the longer the mice are followed, the growth was steepest between follow-up time periods of 1 and 5 weeks after initiation of drug administration. After 5 weeks duration of follow-up time, the increase in power started to plateau with additional follow-up (Figure 3C). Additionally, there was not a large increase in power between measuring tumors 2 times a week versus 3 times a week, especially under the conditions of longer follow-up durations.

### Power under combinations of settings

Power under combinations of the treatment effect size, inter-mouse variation, and follow-up schedule is shown in Table 2. Under all settings, there was sufficient power for a large treatment effect size. When there was a medium treatment effect, the only combination which yielded power above 0.8 was under the specific conditions of a small amount of variation, follow-up of 8 weeks, and alpha that was set to .2. No conditions showed sufficient power under the assumption of a small treatment effect.

**Table 2.** Calculated statistical power under test conditions.

	Mouse variation	α = .05			α = .20		
		Small ${\hat{\beta }}_{Effect}$	Medium ${\hat{\beta }}_{Effect}$	Large ${\hat{\beta }}_{Effect}$	Small ${\hat{\beta }}_{Effect}$	Medium ${\hat{\beta }}_{Effect}$	Large ${\hat{\beta }}_{Effect}$
4 wk							
Twice weekly	Average ${\hat{\tau }}_{1}$	0.09	0.25	**0.82**	0.25	0.5	**0.94**
	Small ${\hat{\tau }}_{1}$	0.12	0.38	**0.96**	0.32	0.65	**0.99**
Three times weekly	Average ${\hat{\tau }}_{1}$	0.10	0.28	**0.86**	0.27	0.54	**0.96**
	Small ${\hat{\tau }}_{1}$	0.15	0.46	**0.99**	0.35	0.72	**1.00**
8 wk							
Twice weekly	Average ${\hat{\tau }}_{1}$	0.12	0.35	**0.94**	0.3	0.62	**0.99**
	Small ${\hat{\tau }}_{1}$	0.23	0.71	**1.00**	0.48	**0.89**	**1.00**
Three times weekly	Average ${\hat{\tau }}_{1}$	0.12	0.36	**0.95**	0.31	0.62	**0.99**
	Small ${\hat{\tau }}_{1}$	0.24	0.74	**1.00**	0.49	**0.91**	**1.00**

Mice are measured for 4 or 8 weeks either twice a week (8/16 measurements), or 3 times a week (12/24 measurements). Mouse variation is represented by $\tau_{1}$ , either the median from the cohort or the first quartile. Treatment effect size is represented by ${\hat{\beta }}_{\mathrm{Effect}}$ , is either the median, Q1, or Q2 from the cohort. Effects detectable that exceed a power of 80% are bolded.

### Required sample size to reach 80% power

The number of mice required per group to reach 80% power is listed in Table 3. At both the alpha levels of .05 and .2 there were situations where 1 mouse per group was found to be sufficient. The highest number of mice required for at least 80% power was 21 mice per group for a small effect size and 5 mice per group for a medium effect size.

**Table 3.** Replicate sample size required for 80% power.

	Mouse variation	α = .05			α = .20		
		Small ${\hat{\beta }}_{Effect}$	Medium ${\hat{\beta }}_{Effect}$	Large ${\hat{\beta }}_{Effect}$	Small ${\hat{\beta }}_{Effect}$	Medium ${\hat{\beta }}_{Effect}$	Large ${\hat{\beta }}_{Effect}$
4 wk							
Twice weekly	Average ${\hat{\tau }}_{1}$	21	5	1	12	3	1
	Small ${\hat{\tau }}_{1}$	13	3	1	7	2	1
Three times weekly	Average ${\hat{\tau }}_{1}$	18	5	1	11	3	1
	Small ${\hat{\tau }}_{1}$	10	3	1	6	2	1
8 wk							
Twice weekly	Average ${\hat{\tau }}_{1}$	14	4	1	8	2	1
	Small ${\hat{\tau }}_{1}$	6	2	1	4	1	1
Three times weekly	Average ${\hat{\tau }}_{1}$	14	4	1	8	2	1
	Small ${\hat{\tau }}_{1}$	5	2	1	3	1	1

Calculations per treatment group to reach 80% power. Where mice measurements were simulated at frequencies of 4 or 8 weeks either twice a week (8/16 measurements), or 3 times a week (12/24 measurements). Mouse variation is represented by $\tau_{1}$ , either the median from the cohort or the first quartile. Treatment effect size is represented by ${\hat{\beta }}_{\mathrm{Effect}}$ , is either the median, Q1, or Q2 from the cohort.

## Discussion

In studies evaluating anti-cancer effects using PDX models, typically vehicle/control and drug treatment groups are represented by a number of mouse replicates. In this work, we examined 4 measures that can impact the power of a 1 × 1 × 1 PDX-designed study: treatment effect size, inter-mouse variation, measurement frequency, and study duration. We utilized simulations as well as a power formula to generate the power calculations; these 2 methods gave equivalent results with respect to determining under which conditions the power is considered sufficient, as defined by greater than 80%.

We found that the factor with the biggest impact is the treatment effect size. When the treatment was very effective, the power could be sufficient (ie, above 80%) when using a 1 × 1 × 1 design. However, when designing a drug efficacy study for a novel agent, initially the size of the treatment effect is unknown. A large effect size should only be assumed if the investigator has adequate evidence from prior/pilot studies. From our PDX studies, the majority of experiments that were run had a measured effect size that led to inadequate power (ie, less than 80%). Gao et al reported similar findings, where most studies were largely considered non-responders. Of 404 treatment models in their study, 303 were considered to have progressive disease, 69 had stable disease, 45 had a partial response, and only 23 models were considered as complete responders.^14^

We also examined the inter-mouse tumor growth variation which is likely the most difficult measure to control. Power did increase when there was less inter-mouse variation, but not to the same degree as with increased effect sizes. In our lab, the inter-mouse variation is minimized by having each study handled by a single person. In general, we found the inter-mouse variation was consistent across studies performed by different staff (Supplemental Figure 1B). We did not examine intra-mouse variation, but this is another factor that could influence statistical power.

Another way to improve the statistical power is by increasing the frequency of tumor measurements. We found that an additional measurement weekly from 2 times to 3 times slightly increased the statistical power, but measuring for a longer period of time was more effective in improving the statistical power.

When designing a study with an unknown effect size, we recommend having at least 5 mice per group to detect a medium effect size: 5 mice achieved 80% power for an average inter-mouse variation, with 4 weeks of follow-up measuring twice a week.

One limitation of this work was that we only investigated independently 1 model per drug. In the context of drug discovery studies, Guo et al^20^ and Eckel-Passow et al^28^ found increasing the number of models tested for each drug greatly increased the power.

Of all the modifications that can be made to improve the power of a 1 × 1 × 1 study, we found that the most substantial was the effect size which is difficult to accurately estimate prior to study. Thus, using 1 × 1 × 1 drug efficacy design can be useful if one is evaluating anticancer compounds with a potentially large effect during the discovery phase of potentially unexpected drug-model relationships.

## Supplemental Material

sj-docx-1-cix-10.1177_11769351221136056 – Supplemental material for Optimizing Drug Response Study Design in Patient-Derived Tumor Xenografts Click here for additional data file. Supplemental material, sj-docx-1-cix-10.1177_11769351221136056 for Optimizing Drug Response Study Design in Patient-Derived Tumor Xenografts by Jessica Weiss, Nhu-An Pham, Melania Pintilie, Ming Li, Geoffrey Liu, Frances A Shepherd, Ming-Sound Tsao and Wei Xu in Cancer Informatics

sj-xlsx-2-cix-10.1177_11769351221136056 – Supplemental material for Optimizing Drug Response Study Design in Patient-Derived Tumor Xenografts Click here for additional data file. Supplemental material, sj-xlsx-2-cix-10.1177_11769351221136056 for Optimizing Drug Response Study Design in Patient-Derived Tumor Xenografts by Jessica Weiss, Nhu-An Pham, Melania Pintilie, Ming Li, Geoffrey Liu, Frances A Shepherd, Ming-Sound Tsao and Wei Xu in Cancer Informatics
