# Supplementary material for: Optimizing Drug Response Study Design in Patient-Derived Tumor Xenografts
Source: Cancer Inform. 2022 Nov 22;21:11769351221136056. doi: 10.1177/11769351221136056 (PMC9685207; doi:10.1177/11769351221136056)
Supplement: sj-docx-1-cix-10.1177_11769351221136056 – Supplemental material for Optimizing Drug Response Study Design in Patient-Derived Tumor Xenografts [file sj-docx-1-cix-10.1177_11769351221136056.docx]

Supplemental Methods

**Power calculations**

For each condition the statistical power was calculated using a formula and/or with simulations. Simulations were performed by creating two populations of 100,000 growth curves (50,000 treated and 50,000 control).

The features of each population (eg. control growth rate, number of measurements, inter-mouse variation,…) were set by the condition and were the same for both populations, the populations only differed in the treatment effect size. One population was under the null hypothesis (H0), with no difference in the growth rate of treated and controls. The other population was under the alternative hypothesis (H1) with the difference in growth rates of the treated and control were set based on the condition. For each population two random growth rates were selected and a log linear model was fit to assess for a difference in growth rates. The t-statistic, which is correlation with the p-value (the larger the absolute value of the t-statistic, the smaller the p-value), was recorded. Then new pairs of mice were sampled and tested until 10,000 t-statistics were recorded for both populations. The null hypotheses distribution is used to calculate the empirical 2.5% and 10% cut off t-statistic which correspond to α/2 where alpha is 0.05 and 0.2. The empirical cut-offs were then applied to the alternative distribution to specify how many tests out of the 10,000 were statistically significant (Supplemental figure 1).

The statistical power formula used was derived from the power formula for repeated measures mixed effects model (supplemental equation 3) in a simplified form (supplemental equation 4). We rearranged the power formula to determine the number of mice required to reach 80% power under different conditions (supplemental equation 3)

Equation 1. Log-linear mixed effects model (When there are replicates per treatment)

|  | ${log(tumor volume)}_{ij}= \hat{\beta}_{int}+\hat{\beta}_{Day}{Day}_{ij}+\hat{\gamma}\times{Treatment}_{ij}+\hat{\beta}_{Effect}{Treatment}_{ij}\times{Day}_{ij}+\hat{b}_{i0}+\hat{b}_{i1}{Day}_{ij}+ \varepsilon_{ij}$  where  $\hat{b}_{i0}\sim N(0,{\hat{\tau}_{0}}^{2})$  $\hat{b}_{i1}\sim N(0,{\hat{\tau}_{1}}^{2})$  $\varepsilon_{ij}\sim N(0,\hat{\sigma}^{2})$ |  |
| --- | --- | --- |

Where $\hat{\beta}_{int}$ is average log tumor volume at time zero of the control group, $\hat{\beta}_{Day}$ is the average logged growth rate for the control, $\hat{\gamma}$ is the difference in log tumor volume at time zero for the treated vs control $\hat{\beta}_{Effect}$ is the log difference in growth rates for treated vs control, $\hat{b}_{i0}$ is mouse specific log volume at time zero, and $\hat{b}_{i1}$ is mouse specific log growth.${\hat{\tau}_{0}}^{2}$ represents the variation in starting tumor volume across mice, ${\hat{\tau}_{1}}^{2}$ represent the tumor growth variation across mice, and $\hat{\sigma}^{2}$ is the variation within mice. In this model $\hat{\tau}_{01}$ is the correlation between the random slope and intercept. The equation could also be applied for a non-logged growth rate if the rates of tumor growth was linear instead of exponential.

Equation 2. Log-linear effects model (When there are no replicates per treatment, 1x1x1 design)

$${log(tumor volume)}_{ij}= \hat{\beta}_{int}+\hat{\beta}_{Day}{Day}_{ij}\times Day+\hat{\gamma}\times{Treatment}_{ij}+\hat{\beta}_{Effect}\times{Treatment}_{ij}\times{Day}_{ij}+ \hat{\varepsilon}$$

Equation 3. Statistical power for a repeated measures mixed effects model

|  | $Z_{(1-\gamma)}= \sqrt{\frac{N\pi(1-\pi){\beta_{effect}}^{2}}{{\sigma_{\beta}}^{2}}-}Z_{(1-\alpha/2)}$ |  |
| --- | --- | --- |

Where

$${\sigma_{\beta}}^{2}=\sigma^{2}\left\{ \sum_{j=1}^{n} {(t_{j}-\bar{t})}^{2} \right\}^{-1}+{\tau_{1}}^{2}$$

Where $\alpha$ is the significance level, $1-\gamma$ is the power, $\beta_{effect}$ is the effect size, t is the day and $\bar{t}$ is $\frac{1}{n}\sum_{j=1}^{n} t_{j}$, $\pi$ is the proportion in each group, N is the total number of mice, and ${\tau_{1}}^{2}$ is the inter-mouse variation across mice, and $\sigma^{2}$is the growth variation within a mouse.

Equation 4. Statistical power for a 1x1x1 model

$$Z_{(1-\gamma)}= \sqrt{\frac{0.5{\beta_{effect}}^{2}}{{\sigma_{\beta}}^{2}}-}Z_{(1-\alpha/2)}$$

Supplemental Figure 1. Power through simulations. The distribution of t-values from the null hypotheses (H0: No treatment effect) is used as a reference to the alternative hypotheses (H1). The red section of the null hypotheses curve represents the bottom 10% t-value. Values below this point represented in blue on the alternative hypotheses curve are considered significant at the α = 0.2 level of significance. The proportion of the H1 curve below the dotted line is the power. The distance between the curves are correlated with the statistical power **(C)** Calculated statistical power based on real individual experiments. Power is calculated based on a 1x1x1 PDX design for alpha levels (type one error rates) of 0.05 and 0.2.


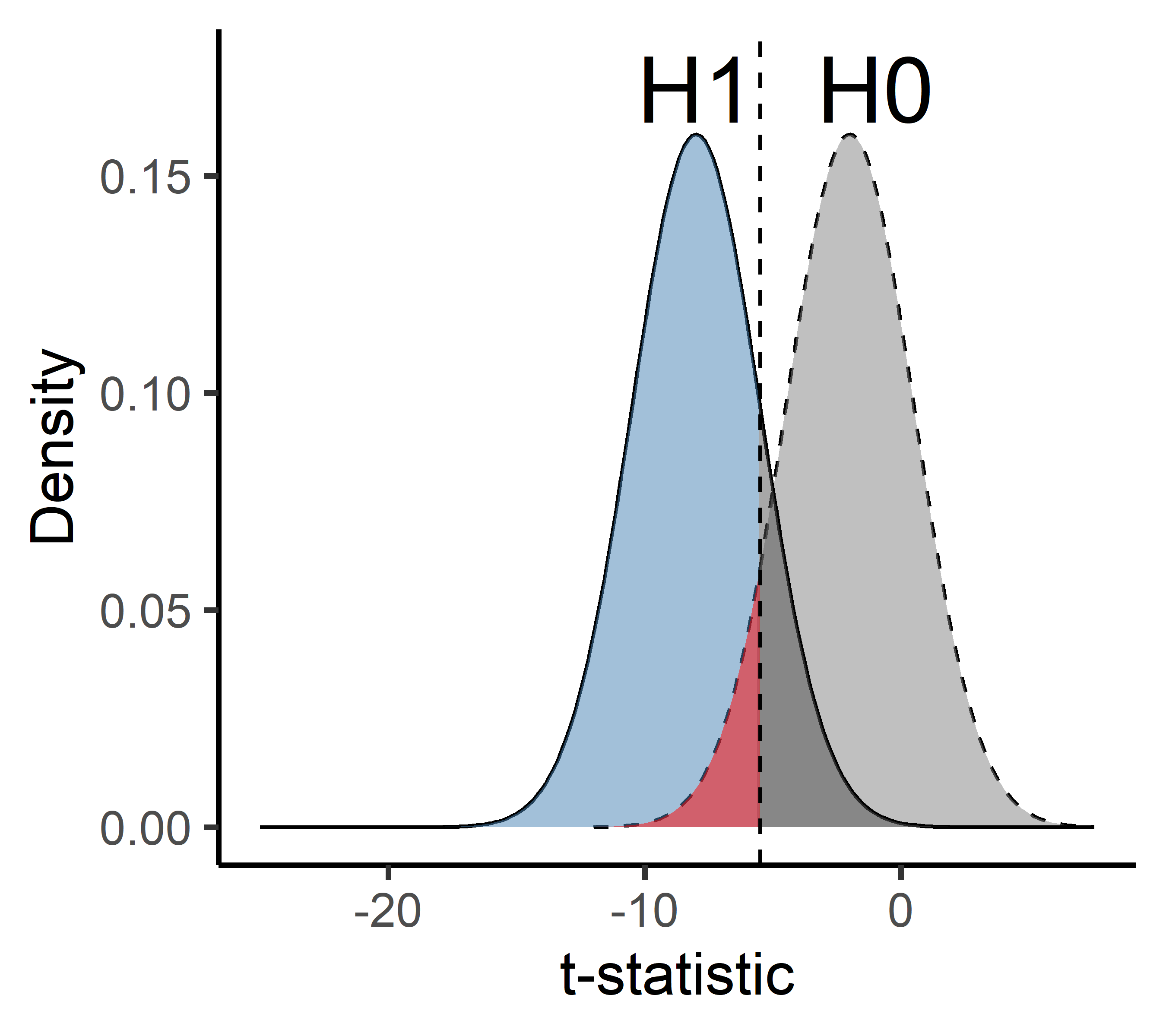


|  |  | \| 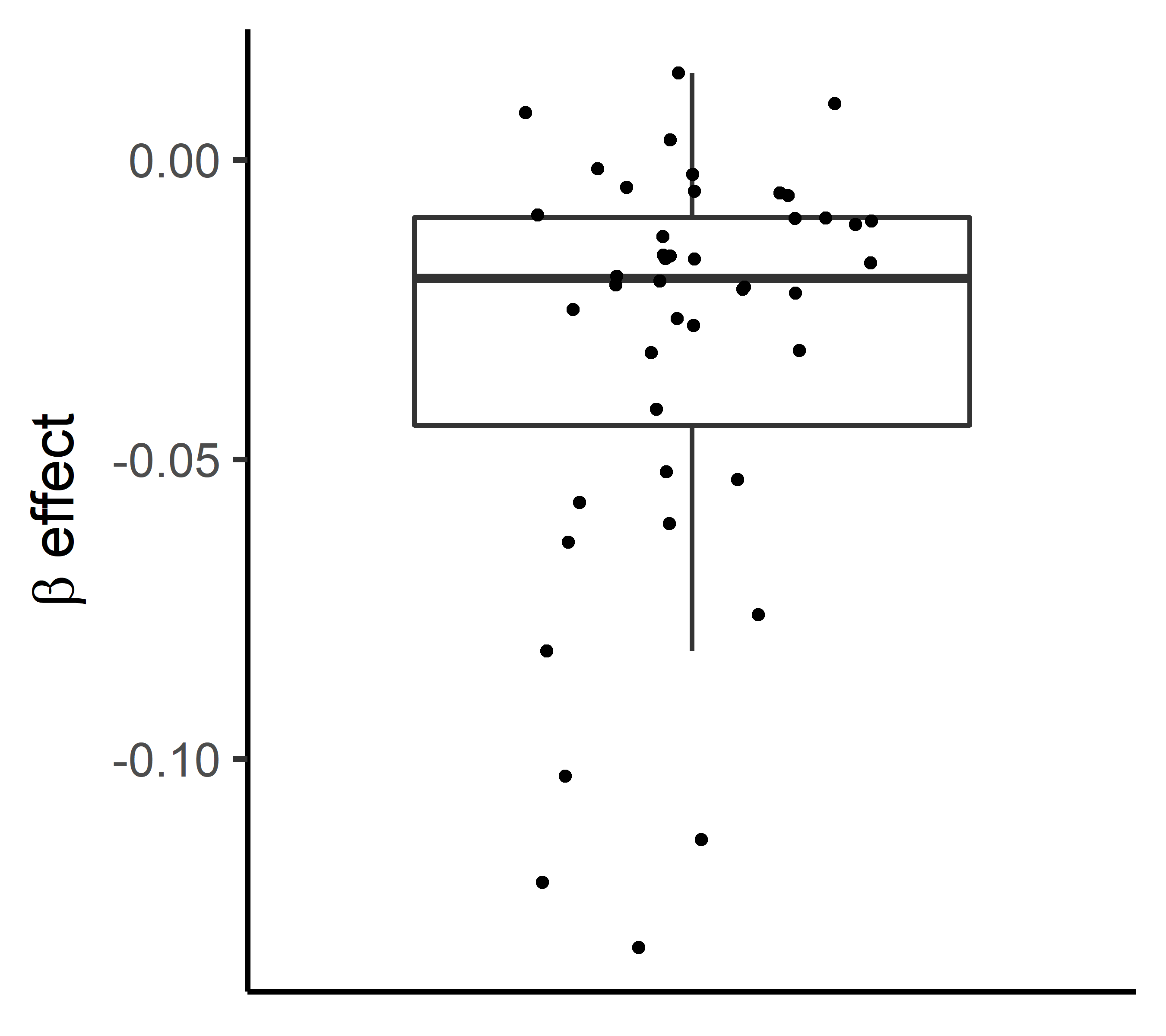  **A** \| \| --- \| \| 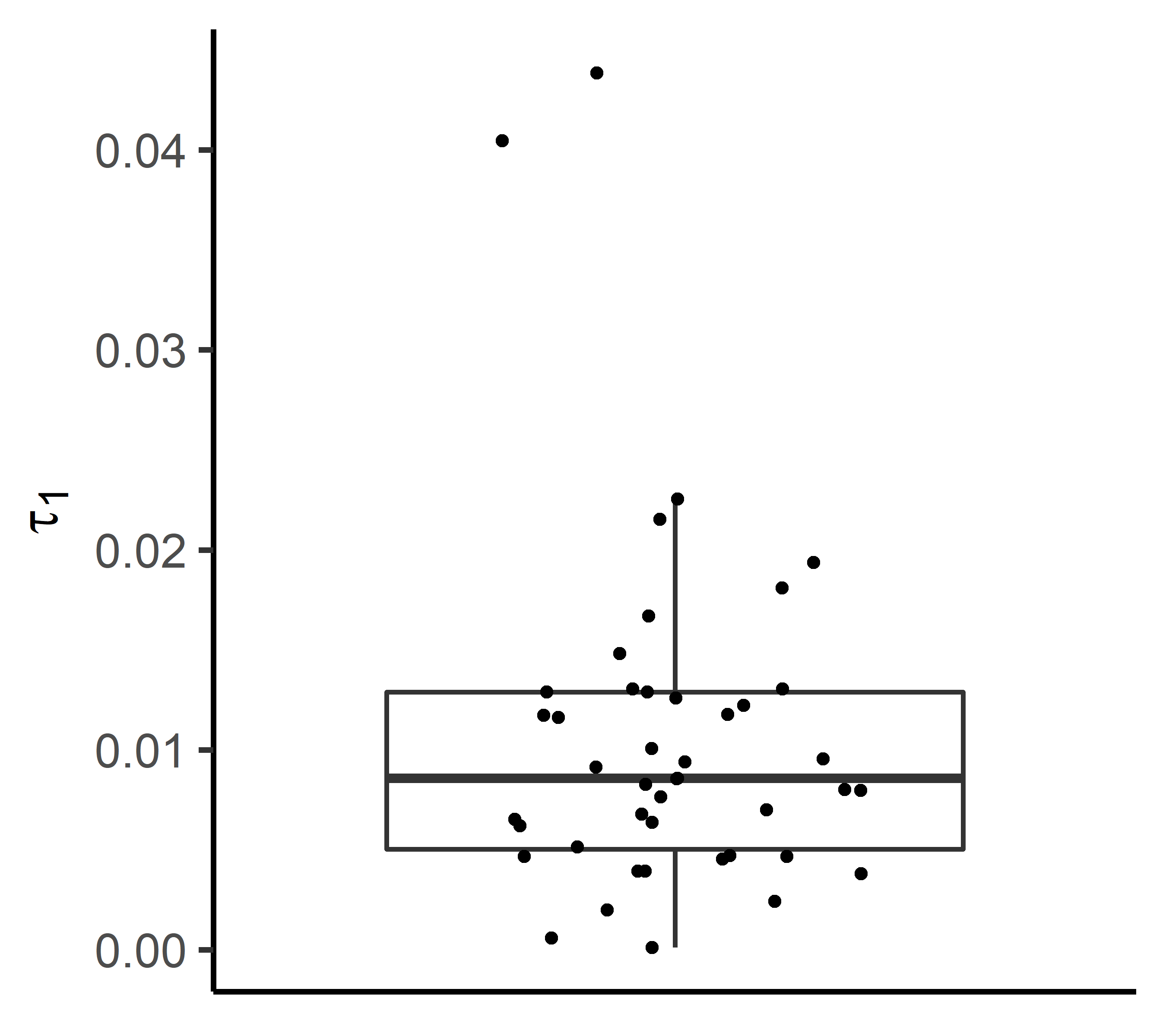  **B** \| |  |
| --- | --- | --- | --- | --- | --- |

Supplemental Figure 2. Distribution of coefficients from log linear mixed effects models from 44 real experiments. **A)** $\beta_{Effect}$, the difference in log daily growth of treated vs control. **B)** $\tau_{1}$the standard deviation in the growth rates across mice.

Supplemental Table 1. Summary 44 PDX Studies.

|  | **N = 44**  **Median (Range)** |
| --- | --- |
| Total mice in experiment  (Treatment + Control) | 12 (10-19) |
| PDX mouse passage | 7 (4,19) |
| Number of tumor volume measurements per mouse | 6 (4,18) |
| Duration of experiment (Days) | 19 (6,60) |

Combined experiments include 531 mice. Time is counted at treatment start.

Supplemental Table 2. Summary PDX experiments 14 treatment conditions

| Experiment number | PHLC  (patient sample ID) | Mouse Passage | Treatment | Experiment date | Total number of mice  (Treatment + Control) |
| --- | --- | --- | --- | --- | --- |
| 1 | 402 | 4 | crizotinib | 6-3-2015 | 10 |
| 2 | 402 | 6 | crizotinib | 7-15-2015 | 13 |
| 3 | 228 | 5 | palbociclib | 12-11-2014 | 11 |
| 4 | 410 | 5 | vandetanib | 8-26-2015 | 12 |
| 5 | 470 | 5 | vandetanib | 10-14-2015 | 12 |
| 6 | 77 | 7 | BKM120 | 1-29-2013 | 11 |
| 7 | 140 | 5 | BKM120 | 4-17-2013 | 12 |
| 8 | 142 | 6 | BKM120 | 12-27-2012 | 11 |
| 9 | 188 | 6 | BKM120 | 12-24-2012 | 10 |
| 10 | 108 | 8 | vandetanib | 5-29-2015 | 10 |
| 11 | 148 | 6 | erlotinib | 7-7-2012 | 11 |
| 12 | 148 | 8 | afatinib | 4-5-2013 | 10 |
| 13 | 148 | 11 | erlotinib | 9-30-2013 | 13 |
| 14 | 164 | 13 | dacomitinib | 7-17-2012 | 14 |
| 15 | 164 | 15 | dacomitinib | 3-4-2013 | 10 |
| 16 | 164 | 16 | dacomitinib | 5-23-2013 | 12 |
| 17 | 164 | 17 | dacomitinib | 8-19-2013 | 12 |
| 18 | 164 | 19 | dacomitinib | 12-6-2013 | 15 |
| 19 | 164 | 8 | dacomitinib | 1-8-2015 | 19 |
| 20 | 192 | 8 | erlotinib | 4-17-2013 | 12 |
| 21 | 200 | 6 | NVP-BGJ398 | 12-24-2013 | 12 |
| 22 | 200 | 7 | crizotinib | 2-20-2014 | 12 |
| 23 | 267 | 7 | NVP-BGJ398 | 9-20-2013 | 12 |
| 24 | 274 | 6 | NVP-BGJ398 | 7-17-2013 | 11 |
| 25 | 274 | 7 | NVP-BGJ398 | 9-26-2013 | 12 |
| 26 | 299 | 7 | NVP-BGJ398 | 11-11-2013 | 12 |
| 27 | 321 | 5 | NVP-BGJ398 | 8-8-2013 | 12 |
| 28 | 321 | 8 | NVP-BGJ398 | 5-1-2014 | 12 |
| 29 | 321 | 9 | crizotinib | 9-15-2014 | 12 |
| 30 | 321 | 10 | AZD4547 | 11-10-2014 | 12 |
| 31 | 110 | 5 | cis + vin | 10-30-2014 | 12 |
| 32 | 181 | 7 | cis + vin | 8-20-2014 | 12 |
| 33 | 191 | 7 | cis + vin | 5-8-2014 | 14 |
| 34 | 119 | 5 | cis + vin | 9-25-2014 | 12 |
| 35 | 229 | 6 | cis + vin | 5-27-2014 | 14 |
| 36 | 235 | 6 | cis + vin | 5-28-2014 | 14 |
| 37 | 235 | 7 | docetaxel | 12-24-2014 | 11 |
| 38 | 235 | 6 | doc + sel concomitant | 8-18-2015 | 12 |
| 39 | 655 | 7 | cis + vin | 4-30-2014 | 12 |
| 40 | 12 | 6 | BKM120 | 6-25-2014 | 12 |
| 41 | 12 | 7 | gefitinib | 9-26-2014 | 12 |
| 42 | 77 | 12 | erlotinib | 2-11-2014 | 10 |
| 43 | 77 | 7 | cetuximab | 7-16-2014 | 13 |
| 44 | 77 | 8 | dacomitinib | 8-26-2014 | 12 |

Cis: cisplatin, Vin: vinorelbine, Sel: selumetinib

*Supplemental Table 2. Estimated coefficients from LMM model for each experiment*

| Experiment number | ${\hat{\boldsymbol{\beta}}}_{\boldsymbol{int}}$ | ${\hat{\boldsymbol{\beta}}}_{\boldsymbol{Day}}$ | $\hat{\boldsymbol{\gamma}}$ | ${\hat{\boldsymbol{\beta}}}_{\boldsymbol{Effect}}$ | $\hat{\boldsymbol{\sigma}}$ | ${\hat{\boldsymbol{\tau}}}_{\boldsymbol{0}}$ | ${\hat{\boldsymbol{\tau}}}_{\boldsymbol{1}}$ | ${\hat{\boldsymbol{\tau}}}_{\boldsymbol{01}}$ | follow up schedule |
| --- | --- | --- | --- | --- | --- | --- | --- | --- | --- |
| 1 | 5.49 | 0.054 | -0.091 | 0.003 | 0.164 | 0.359 | 0.006 | 0.103 | 0,4,7,11,14,18,21,25 |
| 2 | 4.85 | 0.078 | 0.066 | -0.002 | 0.194 | 0.265 | 0.013 | -0.425 | 0,4,7,11,14,18,21 |
| 3 | 5.37 | 0.082 | -0.077 | -0.032 | 0.153 | 0.328 | 0.005 | -0.728 | 0,3,7,10 |
| 4 | 5.71 | 0.073 | -0.094 | -0.064 | 0.122 | 0.239 | 0.013 | -0.332 | 0,3,7,10,14,17 |
| 5 | 5.12 | 0.075 | 0.187 | -0.076 | 0.239 | 0.234 | 0.012 | 0.314 | 0,2,6,9,13,16,20 |
| 6 | 3.88 | 0.054 | 1.120 | -0.017 | 0.148 | 0.772 | 0.023 | 0.514 | 0,3,7,10,14,17,20,23,29 |
| 7 | 4.86 | 0.113 | -0.041 | -0.032 | 0.225 | 0.310 | 0.013 | -0.835 | 0,2,7,10,14 |
| 8 | 5.00 | 0.090 | -0.429 | -0.022 | 0.247 | 0.375 | 0.012 | -0.246 | 0,3,7,11,15,18,21,23 |
| 9 | 5.53 | 0.038 | -0.731 | -0.021 | 0.152 | 0.273 | 0.005 | -0.039 | 0,3,6,9,13,16,20,23,27,30,33,36,42 |
| 10 | 3.42 | 0.128 | 0.027 | -0.009 | 0.156 | 0.374 | 0.007 | -0.673 | 0,4,7,11,14,18 |
| 11 | 4.65 | 0.066 | 0.590 | -0.010 | 0.173 | 0.423 | 0.018 | 0.874 | 0,3,6,10,14 |
| 12 | 4.78 | 0.074 | 0.020 | 0.008 | 0.268 | 0.377 | 0.040 | -0.729 | 0,4,7,11,15,19 |
| 13 | 5.49 | 0.108 | 0.136 | -0.010 | 0.155 | 0.143 | 0.019 | 0.739 | 0,3,7,10 |
| 14 | 4.88 | 0.027 | 0.026 | -0.053 | 0.096 | 0.436 | 0.005 | 0.925 | 0,3,7,10,14 |
| 15 | 4.95 | 0.055 | -0.047 | -0.017 | 0.112 | 0.193 | 0.008 | 0.388 | 0,3,6,9,12,15 |
| 16 | 4.98 | 0.052 | 0.002 | -0.001 | 0.105 | 0.120 | 0.005 | 0.035 | 0,3,7,10,14,17,21,24 |
| 17 | 5.05 | 0.067 | 0.025 | 0.009 | 0.069 | 0.330 | 0.010 | 0.784 | 0,3,7,10 |
| 18 | 5.48 | 0.048 | 0.069 | -0.005 | 0.162 | 0.253 | 0.009 | -0.319 | 0,3,7,10,14,17,21,24 |
| 19 | 5.19 | 0.076 | -0.214 | -0.016 | 0.171 | 0.187 | 0.008 | 0.033 | 0,3,7,10,14,17,21,24,28 |
| 20 | 5.09 | 0.030 | 0.013 | -0.006 | 0.120 | 0.331 | 0.007 | 0.227 | 0,3,6,10,14,17,28,31,35 |
| 21 | 4.94 | 0.076 | 0.049 | -0.027 | 0.109 | 0.032 | 0.022 | 0.979 | 0,3,7,10 |
| 22 | 4.98 | 0.077 | -0.060 | -0.006 | 0.151 | 0.389 | 0.002 | -0.161 | 0,3,7,10,14,17,21,24 |
| 23 | 5.15 | 0.117 | -0.051 | -0.025 | 0.113 | 0.221 | 0.001 | 0.094 | -1,2,6,9,13,16 |
| 24 | 5.67 | 0.050 | 0.017 | -0.010 | 0.110 | 0.247 | 0.004 | 0.016 | 0,3,7,10,15,17,21,24,28,31,35,38 |
| 25 | 5.21 | 0.072 | 0.037 | -0.013 | 0.121 | 0.069 | 0.004 | 0.856 | -1,2,6,9,13,16,20,23,27 |
| 26 | 5.06 | 0.066 | 0.048 | -0.011 | 0.126 | 0.236 | 0.008 | -0.581 | 0,3,7,11,14,17,21 |
| 27 | 5.43 | 0.085 | 0.058 | -0.016 | 0.557 | 0.006 | 0.000 | -0.003 | 0,3,7,10,14 |
| 28 | 5.08 | 0.142 | -0.106 | -0.061 | 0.155 | 0.435 | 0.010 | -0.973 | 0,4,7,11 |
| 29 | 5.26 | 0.087 | -0.101 | -0.005 | 0.147 | 0.351 | 0.002 | 0.359 | 0,4,7,11,14 |
| 30 | 5.17 | 0.072 | -0.199 | -0.017 | 0.181 | 0.171 | 0.013 | 0.508 | 0,3,7,10,14,17,21 |
| 31 | 5.24 | 0.038 | -0.187 | -0.028 | 0.137 | 0.236 | 0.009 | 0.292 | 0,1,4,8,11,15,22,25,29,32,36,39 |
| 32 | 4.25 | 0.234 | 0.074 | -0.057 | 0.282 | 0.052 | 0.012 | 0.832 | 0,3,7,10,14,17 |
| 33 | 4.25 | 0.144 | 0.148 | -0.114 | 0.275 | 0.196 | 0.009 | 0.514 | 2,6,9,12,15,19 |
| 34 | 5.46 | 0.181 | -0.053 | -0.082 | 0.143 | 0.287 | 0.005 | 0.378 | 0,3,7,10 |
| 35 | 5.30 | 0.117 | -0.050 | -0.103 | 0.170 | 0.412 | 0.012 | 0.298 | -1,2,6,9,12,16 |
| 36 | 5.05 | 0.106 | 0.178 | -0.021 | 0.175 | 0.450 | 0.009 | -0.926 | -1,2,6,9,12,16 |
| 37 | 5.54 | 0.093 | -0.322 | 0.014 | 0.127 | 0.324 | 0.008 | -0.064 | 1,5,8,12,15,19 |
| 38 | 5.18 | 0.083 | 0.013 | -0.121 | 0.214 | 0.324 | 0.006 | -0.684 | 1,4,8,11,15,18,22,25,29 |
| 39 | 5.41 | 0.130 | -0.015 | -0.052 | 0.271 | 0.506 | 0.017 | 0.904 | -1,2,6,9 |
| 40 | 4.43 | 0.039 | -0.314 | -0.020 | 0.158 | 0.226 | 0.007 | -0.671 | 1,4,8,11,15,18,22,25,29,32,36,39,43  ,46,50,53,57,60 |
| 41 | 5.21 | 0.032 | -0.788 | -0.042 | 0.218 | 0.160 | 0.004 | -0.671 | 0,4,8,11,15,18,22,25,29,32,36,  39,43,46,50 |
| 42 | 5.08 | 0.072 | -0.001 | -0.019 | 0.336 | 0.829 | 0.044 | -0.960 | 0,3,7,8,10,13,15,17 |
| 43 | 5.21 | 0.082 | -0.080 | -0.022 | 0.133 | 0.222 | 0.013 | 0.866 | 0,3,7,10,14,17,21,24,28 |
| 44 | 5.20 | 0.103 | 0.004 | -0.132 | 0.143 | 0.316 | 0.015 | -0.326 | 1,5,8,12 |
